# Foot Placement and Arm Position Affect the Five Times Sit-to-Stand Test Time of Individuals with Chronic Stroke

**DOI:** 10.1155/2014/636530

**Published:** 2014-06-16

**Authors:** Patrick W. H. Kwong, Shamay S. M. Ng, Raymond C. K. Chung, Gabriel Y. F. Ng

**Affiliations:** Department of Rehabilitation Sciences, The Hong Kong Polytechnic University, Hung Hom, Hong Kong

## Abstract

*Objectives*. To investigate the effect of two foot placements (normal or posterior placement) and three arm positions (hands on the thighs, arms crossed over chest, and augmented arm position with elbow extended) on the five times sit-to-stand (FTSTS) test times of individuals with chronic stroke. *Design*. Cross-sectional study. *Setting*. University-based rehabilitation clinic. *Participants*. A convenience sample of community-dwelling individuals with chronic stroke (*N* = 45). *Methods*. The times in completing the FTSTS with two foot placements and the three arm positions were recorded by stopwatch. *Results*. Posterior foot placement led to significantly shorter FTSTS times when compared with normal foot placement in all the 3 arm positions (*P* ≤ 0.001). In addition, hands on thigh position led to significantly longer FTSTS times than the augmented arm position (*P* = 0.014). *Conclusion*. Our results showed that foot placement and arm position could influence the FTSTS times of individuals with chronic stroke. Standardizing the foot placement and arm position in the test procedure is essential, if FTSTS test is intended to be used repeatedly on the same subject.

## 1. Introduction

Stroke is a common cause of impaired mobility and disability in daily activity [1–3]. Rehabilitation after stroke usually is a prolonged or even lifelong undertaking for the survivors [4]. In order to document the severity of impairment and to monitor progress in the course of rehabilitation, different outcome measures have been developed.

The five times sit-to-stand (FTSTS) test was designed by Csuka and McCarty in 1985 [5]. It is used to assess the functional muscle strength of the lower limbs, especially with older adults. The subject is instructed to stand up from sitting for five times as quickly as possible without using the hands for support. The total duration is recorded in seconds. The FTSTS is an outcome measure commonly used in stroke rehabilitation [6–11]. The test has been shown to have excellent intrarater reliability (intraclass correlation coefficient [ICC_3,1_] = 0.970 − 0.976), interrater reliability (ICC_3,2_ = 0.999), and test-retest reliability (ICC_2,1_ = 0.994 − 1.000) in individuals with chronic stroke [6]. In addition, good test-retest reliability has also been reported with healthy subjects of different ages [12, 13]. Although Mong and colleagues demonstrated that the FTSTS times had good reliability under their adopted protocol [6], the testing procedures of FTSTS were not well standardized across different clinical studies.

The time for performing the FTSTS has also been found to be negatively correlated with knee flexor strength in both the affected leg (*ρ* = −0.753) and the unaffected leg (*ρ* = −0.830) in individuals with chronic stroke [6]. In addition, the FTSTS times were found to be negatively correlated with lower limb muscle strength among older women [14] and a useful independent predictor of deterioration of ability in the activities of daily living over subsequent 3 years for the elderly [15].

Initial foot placement would affect the distance travelled by the body's centre of gravity (CoG) and leverage in rising from a seat [16–18]. Kawagoe et al. [17] demonstrated that forward displacement of CoG [17] during standing up was significantly longer in normal foot placement when compared to posterior foot placement, which was referred to 10 cm behind the normal position. However, initial foot placement was not clearly mentioned in previous studies adopting FTSTS as an outcome measure [6–12, 14, 15, 19–21]. The effect of normal and posterior foot placement on FTSTS times has not been investigated in previous studies.

Result of previous biomechanical study demonstrated that arm position could influence the momentum of upper body generated during sit-to-stand, and restricted arm position would lead to different strategy adopted by the subject when rising up from sitting [22]. However, arm position was not standardized across different clinical studies. Subjects were sometimes instructed to cross their arms in front of the chest [9, 12, 14, 19–21] or to put their hands on their thighs [6]. In some published studies, the arm position was not even mentioned [7, 8, 10, 11, 15]. Augmented arm position was referred to as the position of two hands gripping together with the shoulders flexed at 90° and the elbows fully extended. Although augmented arm position was commonly used in clinical setting to facilitate sit-to-stand movement in subject with stroke [23], the effect of different arm positions including augmented arm position on FTST times has not yet been investigated.

We hypothesized that foot placement and arm position of the subject during FTSTS would lead to significant differences of the FTSTS times in individuals with stroke. The objectives of the present study were to investigate the effect of (1) 2 foot placements (normal and posterior placement) and (2) 3 arm positions (hands on thighs, arms crossed over chest, and augmented arm position with elbow fully extended) on the FTSTS times of individuals with chronic stroke.

## 2. Methods

### 2.1. Participants

Forty-five community-dwelling individuals with chronic stroke were recruited from a local self-help group for stroke survivors. Subjects were included in the study if they (1) were 50 years or above, (2) had experienced a single stroke at least 1 year before the study, and (3) were able to stand up from a chair without any external support. Subjects were excluded if they (1) were unable to follow commands properly, (2) had an Abbreviated Mental Test score below 6 [24], (3) were medically unstable, or (4) were suffering from other neurological or musculoskeletal disorders which could affect sit-to-stand performance.

The Ethics Committee of the administrative institution approved the study protocol. The objectives and procedures of the study were clearly explained to all subjects and they all signed written consent forms. The study procedure followed the guidelines set by the Declaration of Helsinki for human experiments.

### 2.2. Procedure

This study was conducted in a university-based rehabilitation clinic. An armless, height-adjustable chair was used in this study to ensure subjects' hip was in 90 degrees flexion when seated. The subjects were instructed to stand up and sit down from a height-adjustable chair 5 times as quickly as they could. The standardized instruction given for each trial was “on the count of 3, please stand up and sit down 5 times as fast as you can.” The timing started when the subject's back left the back rest and ended when their back touched the back rest after the 5th repetition. The time was recorded by hand using a digital stop watch.

**Figure 1:**
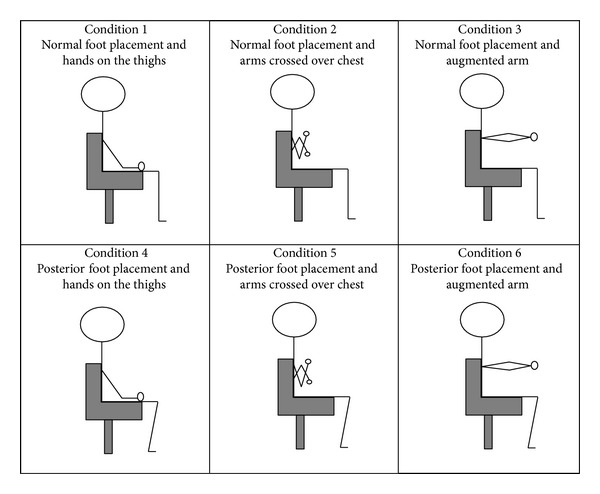
Diagram showing 6 experimental conditions.

The effects of normal and posterior foot placement together with hands on thighs, arms crossed over chest, and augmented arm position on FTSTS times were investigated in this study. Seat height was adjusted according to their lower leg length in all trials. The lower leg length was defined as the perpendicular distance between the fibular head and the floor, when the subject sat on the chair with the knees in 90° of flexion and the ankles in the neutral position. This sitting position was also defined as the normal foot placement. Posterior foot placement was defined as having both heels positioned 10 cm backward from the normal foot placement. The setup was shown in Figure 1.

Each subject was required to perform the FTSTS under 6 experimental conditions in a random sequence by drawing lots. Two trials were performed under each condition, with a 2-minute rest between each trial to avoid fatigue. The 6 experimental conditions were as follows:

 Condition 1: normal foot placement and hands on the thighs;
 Condition 2: normal foot placement and arms crossed over chest;
 Condition 3: normal foot placement and augmented arm;
 Condition 4: posterior foot placement and hands on the thighs;
 Condition 5: posterior foot placement and arms crossed over chest;
 Condition 6: posterior foot placement and augmented arm.

### 2.3. Statistical Analysis

Two-way repeated measures ANOVA were conducted to examine the significance of the observed relationship between 2 foot placements and 3 arm positions with FTSTS times. If the main effects of arm position were statistically significant, post hoc multiple comparison test with Bonferroni adjustment would be used to evaluate the differences of FTSTS times between the 3 arm positions. Null hypothesis will be rejected if *P* < 0.05. All the statistical analysis was conducted with the help of version 16.0 of the SPSS for Windows software package.

## 3. Results

The demographic characteristics of the 45 subjects are shown in Table 1. Their mean age was 60 ± 5.6 years, with an average poststroke duration of 7.1 ± 2.9 years.  Table 2 shows the average FTSTS times with the different foot placements and arm positions. The mean FTSTS times for the 6 conditions ranged from 15.2 to 17.1 seconds.

Two-way repeated measures ANOVA revealed no significant interaction between foot placement and arm position on the FTSTS times [*F*(2,88) = 0.632, *P* = 0.534]. Both the main effect of foot placement and arm position were statistically significant with *F*(1,44) = 97.69, *P* < 0.001, and *F*(2,88) = 3.873, *P* = 0.024, respectively. The significant main effect of foot placement indicated that the normal foot placement led to a significantly longer FTSTS time than the posterior foot placement. Result of post hoc test showed that the hands on thigh position led to significantly longer FTSTS times than the augmented arm position (*P* = 0.014).

## 4. Discussion

This is the first published study to investigate relationship between foot placement and arm position and the FTSTS times of individuals with chronic stroke. Our results showed that both the foot placement and arm position could affect FTSTS times. Posterior foot placement in combination with augmented arm position associates with faster FTSTS times in individuals with chronic stroke.

The average FTSTS times for the 6 conditions ranged from 15.2 to 17.1 seconds. These averages were comparable to those observed in previous studies that reported individuals with chronic stroke [6, 11]. Weiss and colleagues reported FTSTS times of 19.3 ± 2.4 seconds in individuals with chronic stroke [10], but that study included only 7 subjects with a mean age of 70 ± 2.4 years, who were twenty years older than our subjects.

Bohannon had published a meta-analysis which demonstrated that the normal FTSTS time for healthy individuals aged between 60 and 69 years was 11.4 seconds [25]. It was expected that our subjects with chronic stroke would take longer duration to complete the FTSTS. It might probably be due to stroke-specific impairments such as muscle weakness, poor weight bearing on paretic limb [18], impaired balance [26], and fear of falling [27].

### 4.1. Foot Placement

Consistent with the results of healthy adults that posterior foot placement could increase the speed of sit-to-stand [17], our study also showed that posterior foot placement led to shorter FTSTS times with all 3 arm positions. Kawagoe et al. [17] showed that placing the feet at 10 cm behind the normal foot placement was associated with significantly less anterior and abrupt displacement of the CoG, as well as shorter distance between CoG and point of application (PoA) at liftoff. The PoA was defined as the point where compound vector of ground reaction force acting on. Shorter distance between CoG and PoA could reduce the demand on muscle forces required for forward acceleration and backward braking. Posterior foot placement, therefore, could facilitate the sit-to-stand movement. In another study [28], posterior foot placement resulted in a significantly smaller hip flexion angle (*P* < 0.05) in the preextension phase of sit-to-stand. The smaller hip flexion angle implied a shorter distance that the trunk or upper body segment has to move forward to initiate the action of rising from a chair.

Reduced muscular effort required during rising from the seat when the feet are placed posteriorly could also explain shorter FTSTS times taken in posterior foot placement. Reduced tibialis anterior muscle activation during standing up had been found in posterior foot placement when compared with those of normal foot placement [17]. As tibialis anterior muscle activity provides an anterior rotatory force of shank on ankle to bring the CoG forward and to stabilize the ankle [28]; reduced tibialis anterior muscle activity reflected reduced muscular effort during standing up. In another study, maximum hip extension moment (32.7 ± 12.1 Nm) was found to be reduced when the feet were placed posteriorly by 10 cm when compared with the feet being placed by 10 cm forward (148.8 ± 7.5 Nm) during sit-to-stand.

The minimal detectable changes of FTSTS test was calculated according to published data from the study of Mong and colleagues [6]. Using standard deviation of 7.5 seconds and mean intraclass correlation coefficient (ICC) of 0.997 for test retest reliability, 95% minimal detectable changes would be 1.14 seconds [29]. In average, FTSTS completion with posterior foot placement was 1.67 seconds shorter when compared with normal foot placement. Therefore, the change in FTSTS times was unlikely due to measuring error.

### 4.2. Arm Position

The present results revealed significantly shorter FTSTS times with the augmented arm position than the hands on the thighs position. There was, however, no significant interaction between foot placement and arm position.

The augmented arm position might help to shift the CoG forward more efficiently, which could explain its association with faster FTSTS times. Carr and Gentile conducted a kinematic and kinetic analysis of arm position on sit-to-stand movements with force plate and videotaping on 6 healthy young males [22]. The augmented arm position similar to that of our study was shown to induce larger peak CoG momentum in both the horizontal and vertical directions than the restricted arm position. Carr and Gentile explained that the augmented arm position could generate greater propulsive force during a sit-to-stand maneuver [22]. As there is only one study examining the effects of arm positions on sit-to-stand maneuver, further biomechanical studies are warranted.

*Limitations.* This study has several limitations. The population studied was limited to stroke survivors; these results could not be generalized to other populations. Increased sample size and subjects with different degrees of stroke-specific impairment would improve the generalizability of the conclusions. In addition, only two foot placements and three arm positions were studied. A previous study [30] has shown that different foot placement including spontaneous and asymmetric and symmetric foot placement would significantly affect the electromyographic activities of lower limb muscles. Whether other foot placements or arm positions could induce a greater effect on FTSTS times needs further investigation. Further study of the actual kinetics and kinematics of FTSTS with different arm positions is warranted. In view that our study design was cross-sectional, no causal relationships can been established.

All subjects were required to perform FTSTS in 6 different conditions; certain degrees of learning and fatigue effects might affect our results. However, randomization of testing sequences by drawing lots and adoption of 2-minute rest periods would help to minimize the learning and fatigue effects.

## 5. Conclusions

Posterior foot placement and augmented arm position were found to associate with shorter FTSTS times for individuals with chronic stroke. The study did not aim to identify an optimal starting position for the FTSTS test, but the results did highlight the fact that foot placement and arm position had a significant influence on the FTSTS times of stroke survivors. If the test is to be repeated with the same subject, standardizing the arm and foot positions in the test procedure are essential in clinical and research setting.

## Figures and Tables

**Table 1:** Descriptive characteristics of the subjects (*n* = 45).

Variables	*n* (%)
Gender (male/female)	32 (71)/13 (29)
Side of hemiplegia (right/left)	25 (56)/20 (44)
Cause of stroke (ischemic/hemorrhagic)	28 (62)/17 (38)

Variables	Mean ± SD (ranges)

Age (y)	60 ± 5.6 (50–70)
Height (m)	1.6 ± 6.8 (1.4–1.7)
Body weight (kg)	66.6 ± 10.1 (41–93)
BMI (kgm^−2^)	25.7 ± 3.1 (20.6–34.6)
Years poststroke (y)	7.1 ± 2.9 (2.4–16.9)

BMI: body mass index.

**Table 2:** Average FTSTS times with different foot placements and arm positions.

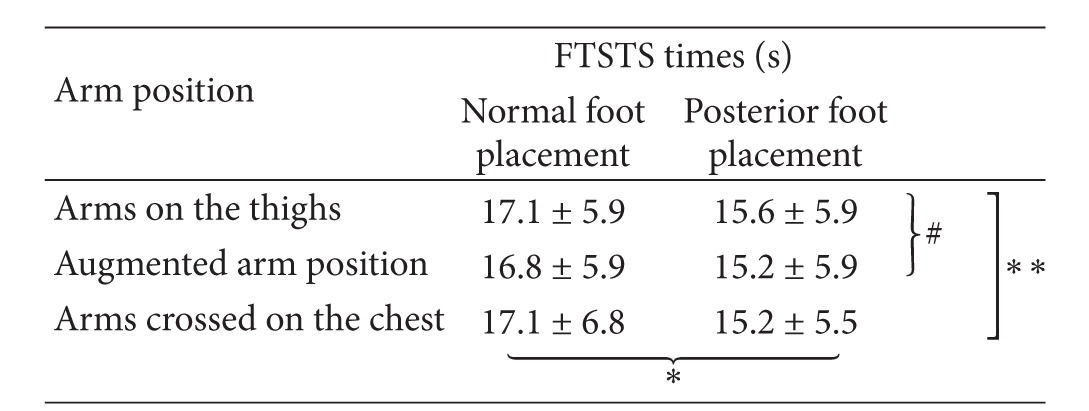

*Significant main effect of foot placements (*P* < 0.001).

∗∗Significant main effect of different arm positions (*P* = 0.024).

^#^Significant difference between arm positions (*P* = 0.014).
